# Gold nanoparticle decorated dithiocarbamate modified natural boehmite as a catalyst for the synthesis of biologically essential propargylamines†

**DOI:** 10.1039/d2ra03725d

**Published:** 2022-11-07

**Authors:** Elham Zarenezhad, Reza Taghavi, Parisa Kamrani, Mojtaba Farjam, Sadegh Rostamnia

**Affiliations:** Noncommunicable Diseases Research Center, Fasa University of Medical Sciences Fasa Iran el.zarenezhad@fums.ac.ir; Organic and Nano Group (ONG), Department of Chemistry, Iran University of Science and Technology (IUST) PO Box 16846-13114 Tehran Iran rostamnia@iust.ac.ir

## Abstract

Here, we prepare an Au NP decorated dithiocarbamate functionalized boehmite (γ-AlO(OH)@C-NHCS_2_H·Au_NPs_). This stepwise synthetic method gives an efficient, cost-effective, and green heterogenous Au-based nanocatalyst for the A^3^-coupling preparation of the biologically essential propargylamines. Different characterization methods, including FT-IR, XRD, SEM, TEM, EDX spectra, and elemental SEM-mapping, were employed to investigate the structure of the manufactured γ-AlO(OH)@C-NHCS_2_H·Au_NPs_. Then we used the prepared composite as a heterogeneous gold-based nanocatalyst for the one-pot A^3^-coupling preparation of propargyl amines by reacting a variety of aldehydes, amines, and phenylacetylene which exhibited promising results.

## Abstract 2

Here, we prepare an Au NP decorated dithiocarbamate functionalized boehmite (γ-AlO(OH)@C-NHCS_2_H·Au_NPs_).

## Graphical abstract

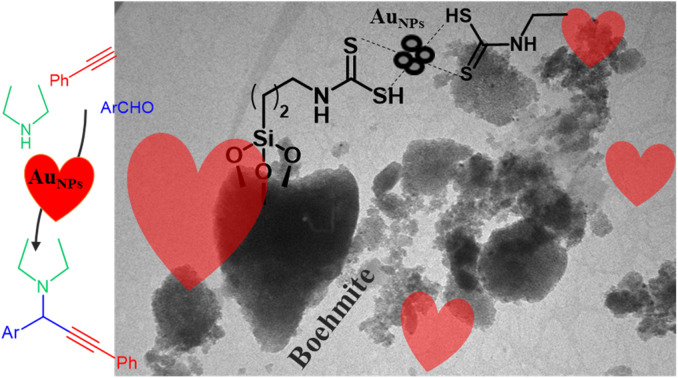

## 1 Introduction

**Scheme 1:**
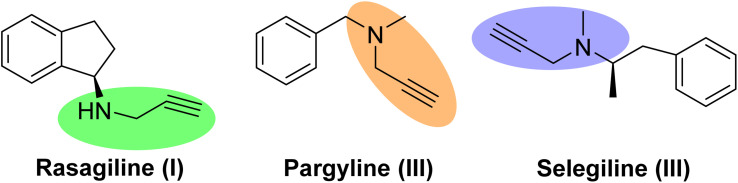
Example of approved drugs having a propargylamines framework.

The term A^3^-coupling reaction refers to the one-pot three-component reaction of an aldehyde, an amine, and a terminal alkyne in the presence of a transition metal to yield propargyl amines. Due to the broad application of such compounds in organic synthesis, especially as intermediates for preparing medicinally essential compounds, the A^3^-coupling preparation of propargyl amines has attracted a great deal of attention in the past two decades, and considerable progress has been achieved.^1,2^ This class of compounds is widely applied toward neurodegenerative disorders such as Alzheimer's and Parkinson's diseases. Many approved drugs, including rasagiline (I), pargyline (II), and selegiline (III) have a propargylamine framework. Such compounds possess monoamine oxidase (MAO) inhibitory properties, and the use of these compounds has been reported in mitochondrial protection and providing neuronal properties (Scheme 1).^3–5^ Utilization of metal NPs helps the progress of this reaction by increasing the acidity of the C–H bond of the terminal alkyne.^6^ To date, various transition metal NPs such as Au, Cu, and Ag have been employed to promote this reaction.^7–9^

Recently, gold NPs were applied as catalysts to promote various reactions, including degradation of environmental pollution, hydrogen generation, organic transformation, *etc.*^10–13^ Such efforts help to disbelief the traditional idea that gold suffers from the lack of catalytic properties. There are various reports on preparing propargyl amines using Au NPs as catalysts.^14–17^ Gold NPs increases the selectivity and yield of the preparation of propargyl amines due to their small size, high surface-to-volume ratio, and intrinsic properties.^6^ The catalytic properties of the gold NPs are directly influenced by their size and shape. When the gold NPs grow to the micro size regime, their catalytic properties are entirely quenching.^18^ Therefore, various supports were employed to prevent the aggregation of gold NPs.

The critical factor for preparing stable metallic NPs and discovering their catalytic properties was using supports and stabilizers.^19–21^ To date, different supports, such as metal–organic frameworks, nanocrystalline magnesium oxide, alumina, cerium oxide, magnetic NPs, *etc.*, are employed for stabilization and modification of the catalytic performance of Au NPs for the A^3^-coupling reaction.^7,22–25^ A metal NP supported on a nano-size heterogenous support benefits from the high selectivity, preventing the aggregation of the metal NPs, high dispersion in a liquid medium, and high reusability.^18^

Boehmite (γ-AlO(OH)), as a binary layer derivative of aluminum, finds its way into heterogeneous catalyst fields due to its insolubility, inexpensive precursors, commercial availability, non-toxicity, corrosion resistance, high chemical and mechanical stability, high dispersity, and accessible surface modification.^26,27^ The layers of boehmite are connected through hydrogen bonding, and a high-density layer of hydroxyl groups covers its surface. Such structure allows easy modification of its surface for desired applications.^26^

In this report, we modified the surface of the commercially available natural boehmite with the APTES ligand. Then, –NH_2_ modified boehmite was further mutated to the dithiocarbamate functional group decorated composite by adding CS_2_ and was utilized to support the stabilization of Au NPs. The resulting γ-AlO(OH)@C-NHCS_2_H·Au_NPs_ catalyst was used as a heterogeneous catalyst for A^3^-coupling preparation of propargyl amines, which exhibited excellent results.

## 2 Experimental

### 2.1 Synthesis of γ-AlO(OH)@C-NHCS_2_H nanoparticles

In a 100 mL round-bottom flask, to 40 mL anhydrous toluene solution of boehmite (2 g), 4 mL of (3-aminopropyl) triethoxysilane (APTES) was added dropwise. The obtained solution was heated to 100 °C and stirred for 48 h. γ-AlO(OH)@C-NH_2_ was washed with H_2_O and dried in an oven overnight. Next, γ-AlO(OH)@C-NH_2_ was dispersed in 20 mL ethanol under sonication for 10 min 2.2 mL CS_2_ was added to the above solution and stirred for another 12 h. Dithiocarbamate functionalized boehmite (γ-AlO(OH)@C-NHCS_2_H) was washed with ethanol several times and dried at 70 °C overnight.

### 2.2 Immobilization of Au_NPs_ onto the surface of γ-AlO(OH)@C-NHCS_2_H

Immobilization of Au nanoparticles onto the surface of dithiocarbamate functionalized Boehmite (γ-AlO(OH)@C-NHCS_2_H) was performed based on the reported method in the literature.^28^ Generally, 0.3 g of γ-AlO(OH)@C-NHCS_2_H was added to 10 mL of 0.12 M aqueous solution of HAuCl_4_ gradually under sonication. This solution kept stirring for 24 h. The heterogeneous catalyst was separated from the solution by filtration and washed with ethanol (EtOH) for several times to gain γ-AlO(OH)@C-NHCS_2_H·AuCl_3_. Finally, 5 mL of freshly prepared NaBH_4_ in methanol (0.2 mol L^−1^) was added dropwise to the mixture of as prepared γ-AlO(OH)@C-NHCS_2_H·AuCl_3_. This solution was stirred for another 30 min, then filtered and washed with methanol to obtain pure γ-AlO(OH)@C-NHCS_2_H·Au_NPs_.

### 2.3 General synthesis of propargyl amines

In a general process, one mmol of aromatic aldehyde, 1.2 mmol of amine, 1.3 mmol of phenylacetylene, and 0.4 g of γ-AlO(OH)@C-NHCS_2_H·Au_NPs_ as a heterogeneous catalyst, and one mmol of Cs_2_CO_3_ as a base were added to 4 mL of CHCl_3_ at 60 °C and stirred for an appropriate time (Table 2). Next, the heterogeneous catalyst was removed from the solution mixture simply by filtration. Finally, the reaction solvent was evaporated, and corresponding propargyl amines were purified by plate-chromatography. The resulting propargyl amines were investigated by ^1^H NMR and ^13^C NMR (ESI†).

## 3 Results and discussion

**Scheme 2:**
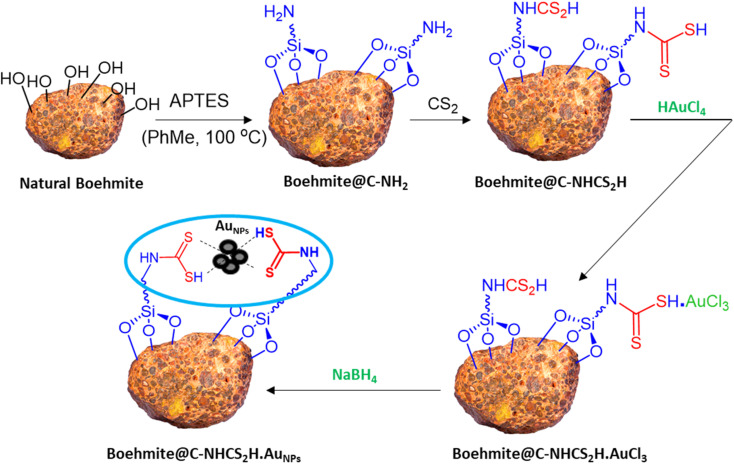
Schematic presentation of the immobilization of Au onto the surface of the γ-AlO(OH)@C-NHCS_2_H.

Due to our interest in developing green and sustainable methodologies for various organic synthesis reactions, herein we report a facile and efficient synthesis of propargyl amines *via* A^3^-coupling reaction using aldehyde, amine, and alkyne in the presence of the γ-AlO(OH)@C-NHCS_2_H·Au_NPs_ nanocatalyst. First, natural boehmite (γ-AlO(OH)) was functionalized with the APTES to give an amine-rich surface. Then, CS_2_ was added to the amine-functionalized boehmite solution to cover its surface with a skin of dithiocarbamate (DTC). Finally, Au NPs were stabilized on the surface of the dithiocarbamate-functionalized boehmite to prepare the γ-AlO(OH)@C-NHCS_2_H·Au_NPs_ catalyst. We employed the crafted nanocomposite as a catalyst for the A^3^-coupling reaction of aldehydes and amines with phenylacetylene under optimal conditions. A schematic illustration of the γ-AlO(OH)@C-NHCS_2_H·Au_NPs_ synthesis is shown in Scheme 2.

**Fig. 1:**
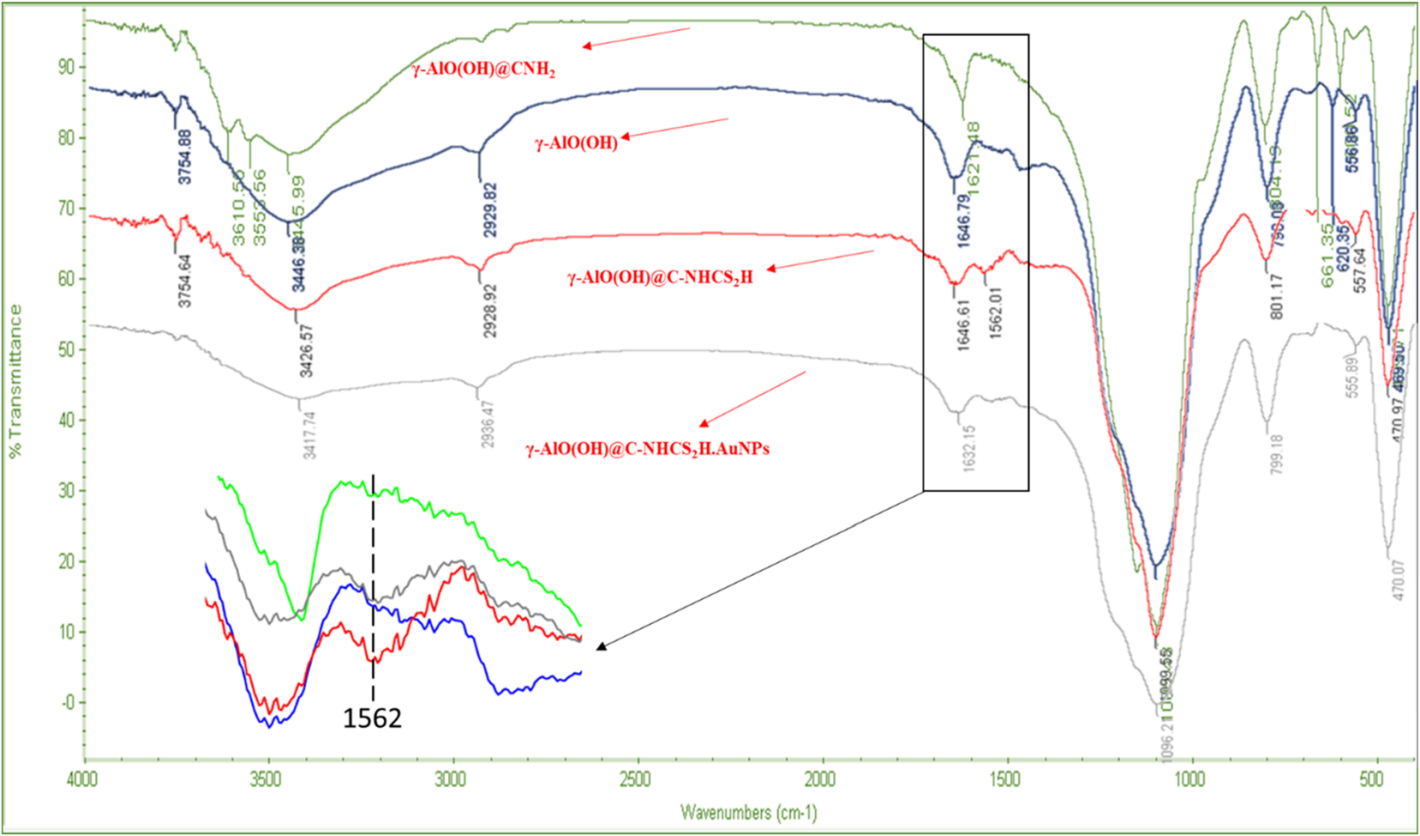
FTIR spectrum of γ-AlO(OH), γ-AlO(OH)C-NH_2_, γ-AlO(OH)@C-NHCS_2_H, γ-AlO(OH)@C-NHCS_2_H·AuNPs.

**Fig. 2:**
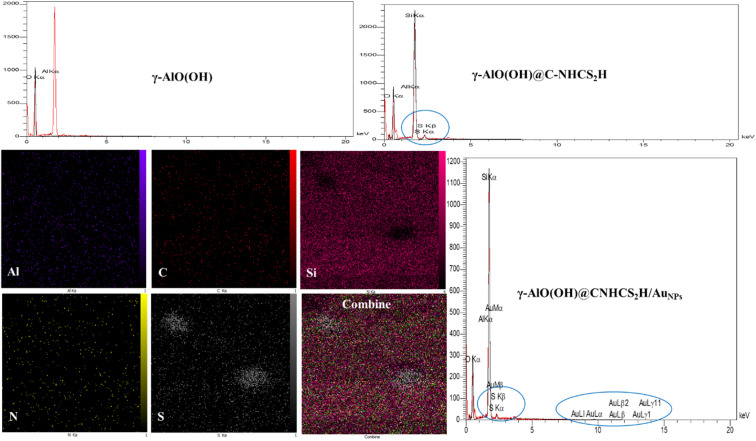
EDX and mapping of γ-AlO(OH), γ-AlO(OH)@C-NHCS_2_H, γ-AlO(OH)@C-NHCS_2_H·AuNPs.

Fig. 1 represents the FT-IR spectra of as-synthesized bare γ-AlO(OH), γ-AlO(OH)@C-NH_2_, γ-AlO(OH)@C-NHCS_2_H, and γ-AlO(OH)@C-NHCS_2_H·Au_NPs_. For the bare γ-AlO(OH), the peak at 469 regarding octahedral morphology of Al. Bending vibration of Al–OH appeared at 1099 cm^−1^, and the stretching vibration of the OH group linked to the Al showed itself at 3446 cm^−1^. For the γ-AlO(OH)@C-NHCS_2_H, peaks in 2900–3000 cm^−1^ and 3426 cm^−1^ regions, probably regarding C–H of carbon groups of the APTES, while O–H functional groups probably attribute to the surface of boehmite. As shown in the spectra of γ-AlO(OH)@C-NHCS_2_H·Au_NPs_, the characteristic peak of –NH at 1646 cm^−1^ is red shifted to 1632 cm^−1^ due to the surface change of bifunctional hybrid nanomaterials. In this case, the characteristic of C=S groups revealed itself at 1562 cm^−1^, and the broadband of the boehmite covers its other characteristics. These can be proof of the successful synthesis of γ-AlO(OH)@C-NHCS_2_H·Au_NPs_. To further prove the formation of the catalyst, EDX spectra and elemental SEM mapping were used to identify the presence of the elements in its structure (Fig. 2).

To determine the composition and the spatial elemental distribution of the synthesized γ-AlO(OH)@C-NHCS_2_H·Au_NPs_, EDX spectra and elemental SEM-mapping were hired (Fig. 2). The results revealed the characteristic peaks of Al, S, O, Si, and Au atoms in the spectrum, indicating the purity and successful deposition of Au onto the γ-AlO(OH)@C-NHCS_2_H support. wt% of Au in the catalyst was 1.36%. Since the attained results for wt% of Au were within the limit of detection for the EDX technique, atomic absorption spectrometry (AAS) was applied to confirm the accuracy of the obtained data. AAS confirms the result of EDX with a 2% percentage error.

**Fig. 3:**
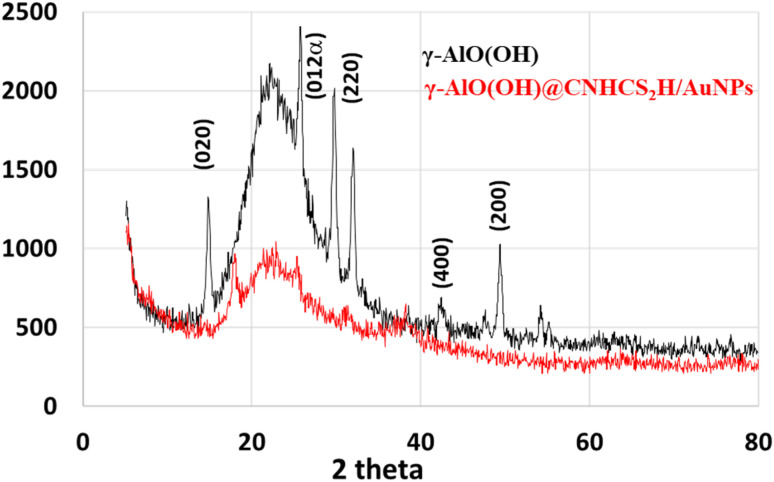
XRD pattern of γ-AlO(OH)@C-NHCS_2_H·Au_NPs_ and γ-AlO(OH).

Fig. 3 represents the XRD pattern of the bare γ-AlO(OH) and γ-AlO(OH)@C-NHCS_2_H·Au_NPs_. The XRD pattern of the bare boehmite shows all the characteristics peaks with a good intensity which specifies its phase purity. The XRD pattern of the modified composite shows less intensity for the characteristic peaks of the boehmite, which is a result of the successful modification of the bare surface of the catalyst. No diffractions were detected for Au NP species from XRD patterns. The reason could be due to the amorphous structure, small size, or low concentration of gold NPs compared to the support (1.36 wt%).^29^

**Fig. 4:**
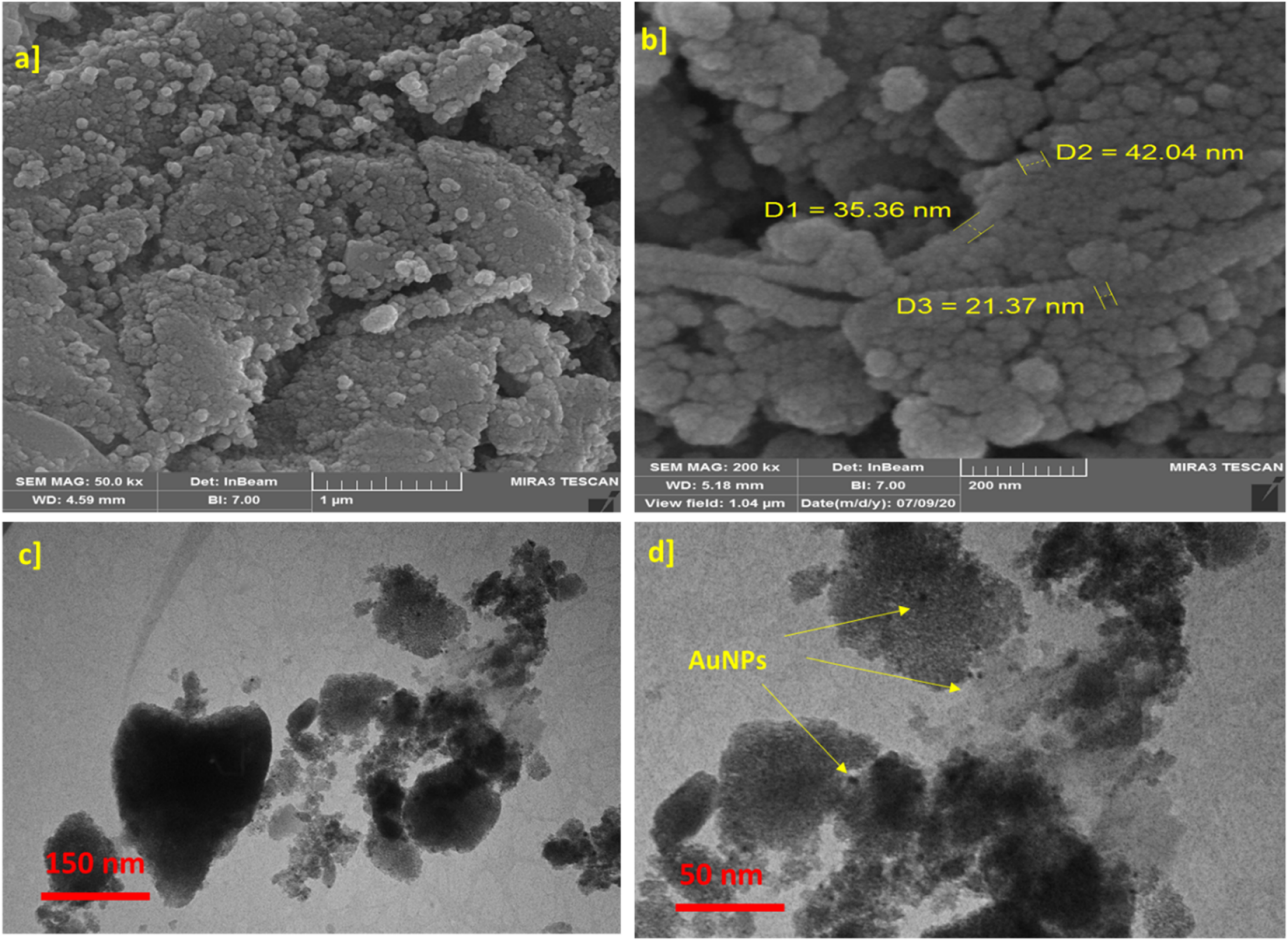
(a) SEM γ-AlO(OH), (b) SEM γ-AlO(OH)@C-NHCS_2_H·Au_NPs_. (c and d) TEM of γ-AlO(OH)@C-NHCS_2_H·Au_NPs_.

FESEM and TEM were employed to investigate the morphology of the synthesized catalyst (Fig. 4). The FESEM image (Fig. 4a) reveals agglomerated morphology of γ-AlO(OH)@C-NHCS_2_H·Au_NPs_ catalyst. The TEM images provide a more detailed insight into the surface of the ensembled catalyst and prove that the functionalization of its surface with dithiocarbamate perfectly prevents aggregation of Au NPs (Fig. 4c and d).

### 3.1 Catalytic performances

We lunch the study of catalytic performance of the γ-AlO(OH)@C-NHCS_2_H·Au_NPs_ toward the A^3^-coupling reaction by using the morpholine, benzaldehyde, and phenylacetylene as the precursors. In this context, we examine the effects of various parameters such as temperature, reaction solvents, reaction time, base, and amount of catalyst to reach the optimum condition. We initiated our study by probing the effect of various solvents on the progress of this reaction (Table 1). Results show that the EtOH and EG solvent restricted the progress of the reaction while other solvents such as acetonitrile and chloroform promoted the A^3^-coupling reaction in good yield. Table 1 indicates that 0.1 mol% of gold NPs onto the γ-AlO(OH)@C-NHCS_2_H·Au_NPs_ in CHCl_3_ solvent gives the most satisfying results (Table 1, entry 5).

**Table 1:** The study results on the effect of various solvents on the A^3^-coupling preparation of propargylamine^a^

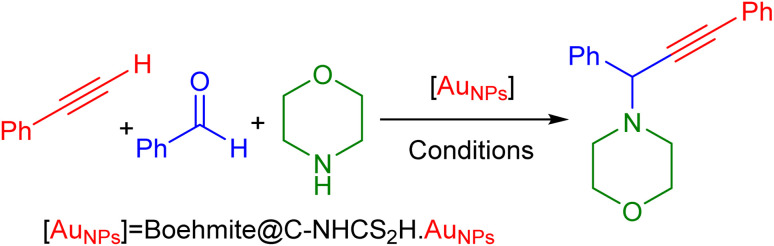						
Entry	Solvent	Base	Temp. (°C)	Cat. (mol%)	Time (h)	Yield^b^ (%)
1	CH_3_CN	KOH	60	0.05	10	75
2	CH_3_CN	KOH	60	0.1	10	75
3	EtOH	KOH	80	0.05	10	45
4	EG	KOH	100	0.05	10	61
5	CHCl_3_	K_2_CO_3_	60	0.1	10	92
6	CHCl_3_	Cs_2_CO_3_	60	0.1	10	96
7	CHCl_3_	KOH	60	0.1	10	87
9	CHCl_3_	NaOH	60	0.1	10	84
10	CHCl_3_	Et_3_N	60	0.1	10	76

a Reaction conditions: 1.2 mmol of morpholine, 1 mmol of benzaldehyde, and 1.3 mmol of phenylacetylene.

b Isolated yields.

**Fig. 5:**
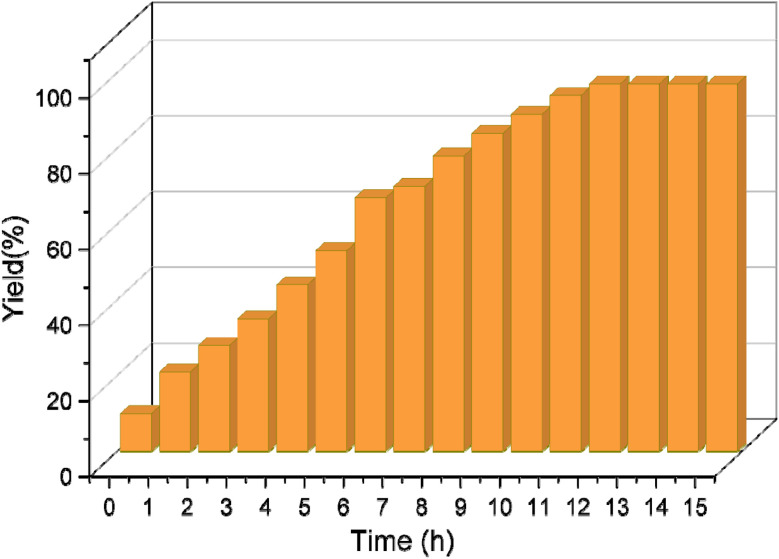
The effect of reaction time on the evolution of A^3^-coupling reaction.

We further investigate the catalytic performance of boehmite@C-NHCS_2_H·Au_NPs_ toward the A^3^-coupling of the model reaction by monitoring the effect of different reaction temperatures and bases. First, we investigate the impact of the temperature on the yield of the reaction. The results indicate that the reaction perfectly proceeds at 60 °C, and a further increase in temperature did not cause considerable changes in the yield of the reaction. Next, we studied the effect of various bases including Et_3_N, NaOH, KOH, K_2_CO_3_, and Cs_2_CO_3_ on the model A^3^-coupling reaction. This study showed that using Cs_2_CO_3_ and K_2_CO_3_ as bases yielded the best results (Table 1, entry 4–5). Fig. 5 represents the results of the studies of the optimum reaction time (0.1 mol% of gold NPs onto the γ-AlO(OH)@C-NHCS_2_H·Au_NPs_, Cs_2_CO_3_ as the base and 60 °C as reaction temperature). Here, the progress of the reaction was investigated by TLC. The maximum yield was achieved after 12 h, as is evident.

**Fig. 6:**
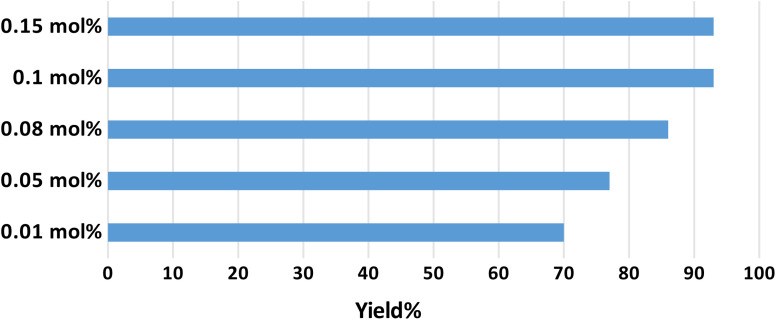
The effect of the mol% of the catalysts on A^3^-coupling reaction.

Optimization of the catalyst amount was performed by testing the catalytic performance of several amounts of γ-AlO(OH)@C-NHCS_2_H·AuNPs in the progress of the model reaction. The results indicate that using 0.1 mol% of γ-AlO(OH)@C-NHCS_2_H·Au_NPs_ (per each mol of the substrate) leads to the maximum yield (Fig. 6).

After optimizing the condition of the model reaction (0.1 mol% of gold NPs onto the γ-AlO(OH)@C-NHCS_2_H·Au_NPs_, Cs_2_CO_3_ as the base and 60 °C as reaction temperature), the generality of the catalyst toward the A^3^-coupling reaction has been tested in the presence of a variety of aldehydes, amines, and phenylacetylene. All the subsequent reactions have been performed at the optimum condition in the presence of Cs_2_CO_3_ as the base. The results of this study are summarized in Table 2. These results indicate that the proposed catalyst can promote the A^3^-coupling reaction for various aldehydes with the electron-donor or electron-withdrawing groups in the ortho or meta position of the aromatic ring. Table 2 also specifies that the reaction's progress is influenced by the electronic characteristics of the aromatic ring of the aldehyde and the yield of the reaction differs from case to case.

**Table 2:** Synthesis of propargylamine derivatives under the optimized conditions^a^

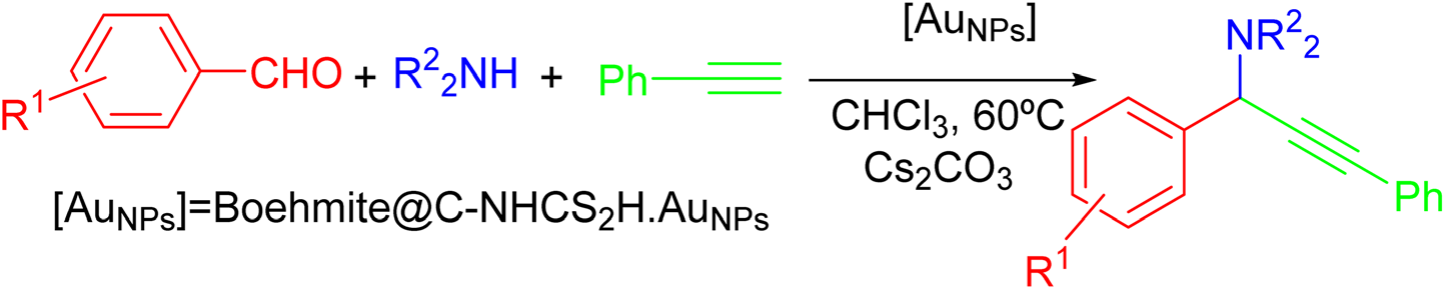				
Entry	Time (h)	R_2_^2^NH	R^1^	Yield^b^ (%)
1	12	Piperidine	H	96
2	15	Morpholine	H	80
3	18	Piperidine	4-Me	75
4	18	Morpholine	4-Me	73
5	16	Piperidine	2-Cl	79
6	18	Morpholine	2-Cl	68
7	18	Morpholine	4-OMe	85
8	16	Piperidine	2-Me	85
9	18	Morpholine	2-Me	73

a Reaction conditions: 1.2 mmol of morpholine, 1 mmol of benzaldehyde, and 1.3 mmol of phenylacetylene.

b Isolated yields in 4 mL of CHCl_3_.

**Fig. 7:**
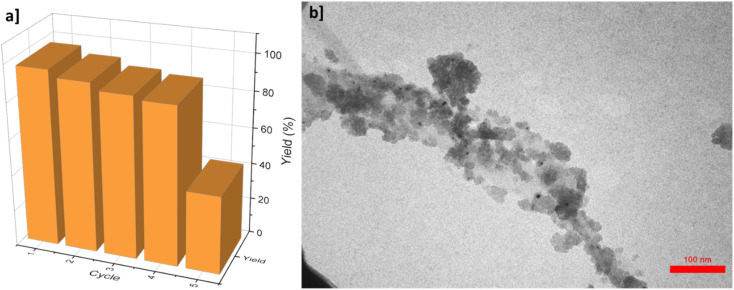
(a) Recyclability of γ-AlO(OH)@C-NHCS_2_H·AuNPs for model A^3^-coupling reaction (b) TEM image of the proposed catalyst after the 4^th^ recycling period.

Reusability of the catalyst is a crucial feature for its scalable applications. To test the reusability of the proposed γ-AlO(OH)@C-NHCS_2_H·AuNPs catalyst for A^3^-coupling reaction of the model reaction, the catalyst was separated from the reaction medium and used for the same reaction at optimum condition for five times. After each run, the catalyst was thoroughly washed with the ethyl acetate several times to ensure the purity of the recycled catalyst, so the reaction kinetic be the same for all the cycles. As shown in Fig. 7a, the yield of the reaction remains almost the same for four cycles but it suddenly drops in the fifth cycle.

Atomic absorption spectrometry (AAS) was employed to track the presence of the Au NPs in the reaction solution, which proves the existence of negligible amounts of the Au NPs in the solution medium, even after four cycles. TEM image of the catalyst after the 4^th^ run reveals its formidable firmness under the reaction conditions (Fig. 7b).

**Scheme 3:**
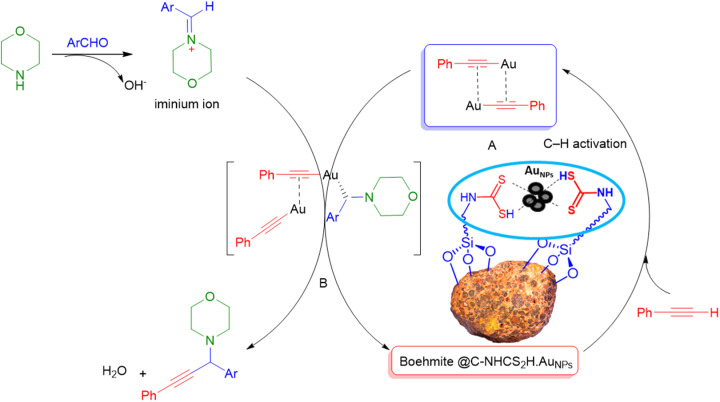
A plausible mechanism for the Au-catalyzed A^3^-coupling.

It is appraises that the γ-AlO(OH)@C-NHCS_2_H·AuNPs catalyst helps the progress of the reaction *via* the Heaney and co-workers mechanism_ENREF_66. At first, gold NPs decorated catalyst helps the activation of C–H of phenylacetylene. Then, coordination of the obtained complex with iminium ion gives an intermediate. Finally, the addition of the alkynylide to iminium ion results in desired propargyl amines (Scheme 3).

## 4 Conclusion

In conclusion, we synthesis an Au NPs decorated dithiocarbamate functionalized boehmite as a cost-effective, green, and efficient heterogeneous catalyst for the one-pot three-component A^3^-coupling preparation of propargyl amines at 60 °C. The γ-AlO(OH)@C-NHCS_2_H·AuNPs catalyst managed to preserve its structure during the preparation procedure and successfully stabilizes monodispersed Au NPs perfectly. The proposed catalyst promotes the preparation of propargyl amines in good yields with a variety of aromatic aldehydes, possessing electron donor and electron-withdrawing groups in different positions of the aromatic ring and showing good recyclability up to four cycles.

## Conflicts of interest

The authors declare no competing financial interest.

## Supplementary Material

RA-012-D2RA03725D-s001

## References

[cit1] Rokade B. V., Barker J., Guiry P. J. (2019). Chem. Soc. Rev..

[cit2] Gholipour B., Shojaei S., Rostamnia S., Naimi-Jamal M. R., Kim D., Kavetskyy T., Nouruzi N., Jang H. W., Varma R. S., Shokouhimehr M. (2021). Green Chem..

[cit3] Manujyothi R., Aneeja T., Anilkumar G. (2021). RSC Adv..

[cit4] Weinreb O., Amit T., Bar-Am O., Youdim M. B. H. (2010). Prog. Neurobiol..

[cit5] Youdim M. B. H., Am O. B., Yogev-Falach M., Weinreb O., Maruyama W., Naoi M., Amit T. (2005). J. Neurosci. Res..

[cit6] Nasrollahzadeh M., Sajjadi M., Ghorbannezhad F., Sajadi S. M. (2018). Chem. Rec..

[cit7] Aghbash K. O., Alamgholiloo H., Pesyan N. N., Khaksar S., Rostamnia S. (2021). Mol. Catal..

[cit8] Liu X., Tan X., Zhou Y., Li Y., Zhang Z. (2019). Res. Chem. Intermed..

[cit9] Akbarzadeh P., Koukabi N. (2019). Appl. Organomet. Chem..

[cit10] Veisi H., Abassi P., Mohammadi P., Tamoradi T., Karmakar B. (2021). Sci. Rep..

[cit11] Mondal S., Sahoo L., Vinod C. P., Gautam U. K. (2021). Appl. Catal., B.

[cit12] Zhu Z., Gao X., Wang X., Yin M., Wang Q., Ren W., Wang B., Lü H., Liao W. (2021). J. Energy Chem..

[cit13] Naixin Kang M. M. M., Wang Q., Djeda R., Wang W., Fu F., Maria de los Angeles Ramirez J.-L. P., Moya S., Coy E., Salmon L., Astruc D. (2020). ACS Appl. Mater. Interfaces.

[cit14] Nourmohammadi M., Rouhani S., Azizi S., Maaza M., Msagati T. A. M., Rostamnia S., Hatami M., Khaksar S., Zarenezhad E., Jang H. W., Shokouhimehr M. (2021). Mater. Today Commun..

[cit15] Lauder K., Toscani A., Scalacci N., Castagnolo D. (2017). Chem. Rev..

[cit16] María G. B., Peters K., Hallett-Tapley G. L., Grenier M., Scaiano J. C. (2013). Chem. Commun..

[cit17] Shore G., Yoo W. J., Li C. J., Organ M. G. (2010). Chem.–Eur. J..

[cit18] Sankar M., He Q., Engel R. V., Sainna M. A., Logsdail A. J., Roldan A., Willock D. J., Agarwal N., Kiely C. J., Hutchings G. J. (2020). Chem. Rev..

[cit19] Alamgholiloo H., Rostamnia S., Hassankhani A., Liu X., Eftekhari A., Hasanzadeh A., Zhang K., Karimi-Maleh H., Khaksar S., Varma R. S., Shokouhimehr M. (2020). J. Colloid Interface Sci..

[cit20] Ahadi A., Alamgholiloo H., Rostamnia S., Liu X., Shokouhimehr M., Alonso D. A., Luque R. (2019). ChemCatChem.

[cit21] Karimi-Maleh H., Alizadeh M., Orooji Y., Karimi F., Baghayeri M., Rouhi J., Tajik S., Beitollahi H., Agarwal S., Gupta V. K., Rajendran S., Rostamnia S., Fu L., Saberi-Movahed F., Malekmohammadi S. (2021). Ind. Eng. Chem. Res..

[cit22] Layek K., Chakravarti R., Lakshmi Kantam M., Maheswaran H., Vinu A. (2011). Green Chem..

[cit23] Corma A., Navas J., Sabater M. J. (2012). Chem.–Eur. J..

[cit24] Abahmane L., Köhler J. M., Grob G. A. (2011). Chem.–Eur. J..

[cit25] Liu L., Tai X., Yu G., Guo H., Meng Q. (2016). Chem. Res. Chin. Univ..

[cit26] Mohammadi M., Khodamorady M., Tahmasbi B., Bahrami K., Ghorbani-Choghamarani A. (2021). J. Ind. Eng. Chem..

[cit27] Paluch P., Potrzebowska N., Ruppert A. M., Potrzebowski M. J. (2017). Solid State Nucl. Magn. Reson..

[cit28] Huang J., Gray D. G., Li C. (2013). Beilstein J. Org. Chem..

[cit29] Jiang H. L., Liu B., Akita T., Haruta M., Sakurai H., Xu Q. (2009). J. Am. Chem. Soc..

