# Supplementary material for: Gold nanoparticle decorated dithiocarbamate modified natural boehmite as a catalyst for the synthesis of biologically essential propargylamines
Source: RSC Adv. 2022 Nov 7;12(49):31680–7. doi: 10.1039/d2ra03725d (PMC9638948; doi:10.1039/d2ra03725d)
Supplement: RA-012-D2RA03725D-s001 [file RA-012-D2RA03725D-s001.pdf]

Electronic Supplementary Material (ESI) for RSC Advances.
This journal is © The Royal Society of Chemistry 2022

Electronic Supporting information

Gold nanoparticle decorated dithiocarbamate modified natural boehmite as a catalyst for the synthesis of biologically essential propargylamines

Elham Zarenezhad, [a] Reza Taghavi, [b] Parisa Kamrani [b], Sadegh Rostamnia [b,*]

[a] *Noncommunicable Diseases Research Center, School of Medicine, Fasa University of Medical Sciences, Fasa, Iran. Email: el.zarenezhad@fums.ac.ir*

[b] *Organic and Nano Group (ONG), Department of Chemistry, Iran University of Science and Technology (IUST), PO Box 16846-13114, Tehran, Iran. Email: rostamnia@iust.ac.ir*

1-(1,3-diphenylprop-2-yn-1-yl)piperidine: ^{1}H NMR ($CDCl_3$, 500 MHz): δ_H (ppm) 1.46 (s, 3H, $NCH_2CH_2CH_2$), 1.60-1.66 (m, 6H, $NCH_2CH_2CH_2$), 2.59 (s, 4H, $NCH_2CH_2CH_2$), 4.83 (s, 1H of NCH), 7.32-7.40 (m, *6H of Ar*), 7.53-7.55 (m, *2H of Ar*), 7.65 (d, $^3J_{HH}$ = 7Hz, 2H).

4-(1,3-diphenylprop-2-yn-1-yl)morpholine: ^{1}H NMR ($CDCl_3$, 500 MHz): δ_H (ppm) 2.66 (s, 2H, NCH_2CH_2O), 3.76 (t, $^3J_{HH}$ = 5.5 Hz, 4H, NCH_2CH_2O), 4.82 (s, 1H of NCH), 7.33-7.41 (m, 6H of Ar), 7.53-7.55 (m, 2H of Ar), 7.64 (d $^3J_{HH}$ = 7.5 Hz, 2H). ^{13}C NMR (125 MHz, CDCl3): δ_c (ppm) 62.03 (NCH), 67.17 (NCH_2CH_2O), 85.03 (Ph-C≡C), 88.48 (HCN-C≡C), 122.97, 127.78, 128.23, 128.31, 128.59, 131.81, 137.79.

4-(3-phenyl-1-(p-tolyl)prop-2-yn-1-yl)morpholine: ^{1}H NMR ($CDCl_3$, 500 MHz): δ_H (ppm) 2.66 (s, 3H of Me), 2.71-2.62 (m, 4H, NCH_2CH_2O), 3.80-3.71 (m, 4H, NCH_2CH_2O), 4.87 (s, 1H of NCH), 7.22-7.19 (m, 2H of Ar), 7.36-7.33 (m, 3H of Ar). ^{13}C NMR (125 MHz, CDCl3): δ_c (ppm)

31.7 (CH_3), 50.7 (NCH_2CH_2O), 61.7 (NCH), 67.9 (NCH_2CH_2O), 85.3 (Ph-C≡C), 88.5 (HCN-C≡C), 123.2, 128.3, 128.4, 128.6, 129.1, 131.7, 135.6, 137.6.

1-(1-(2-chlorophenyl)-3-phenylprop-2-yn-1-yl)piperidine: ^{1}H NMR ($CDCl_3$, 500 MHz): δ_H (ppm) 1.44 (t, $^3J_{HH}$ = 5.5 Hz, 2H, $NCH_2CH_2CH_2$), 1.57-1.61 (m, 4H, $NCH_2CH_2CH_2$), 2.63 (t, $^3J_{HH}$ = 5.5 Hz, 4H, $NCH_2CH_2CH_2$), 5.12 (s, 1H of NCH), 7.25-7.29 (m, 2H of Ar), 7.34 (t, $^3J_{HH}$ = 6.5 Hz, 2H of Ar), 7.51-7.53 (m, 1H of Ar), 7.76-7.77 (m, 1H of Ar). ^{13}C NMR (125 MHz, CDCl3): δ_c (ppm) 24.45 ($NCH_2CH_2CH_2$), 26.14 ($NCH_2CH_2CH_2$), 50.70 (NCH), 59.25 ($NCH_2CH_2CH_2$), 85.81 (Ph-C≡C), 87.63 (HCN-C≡C), 123.17, 126.17, 128.13, 128.27, 128.76.

1-(1-(2-chlorophenyl)-3-phenylprop-2-ynyl)morpholine: ^{1}H NMR ($CDCl_3$, 500 MHz): δ_H (ppm) 2.69 (t, $^3J_{HH}$ = 4.5 Hz, 4H, NCH_2CH_2O), 3.68-3.77 (m, 4H, NCH_2CH_2O), 4.95 (s, 1H of NCH), 7.29-7.36 (m, 5H of Ar), 7.42-7.44 (m, 1H of Ar), 7.51-7.53 (m, 2H of Ar), 7.76 (t, $^3J_{HH}$ = 2.5 Hz, 1H of Ar).
